# Metabolic Syndrome and Chronic Renal Disease

**DOI:** 10.3390/diseases6010012

**Published:** 2018-01-24

**Authors:** Vaia D. Raikou, Sotiris Gavriil

**Affiliations:** 1 Department of Nephrology, Doctors’ Hospital, 26 Kefallinias, 11257 Athens, Greece; 2 Department of Weight-Surgery, Doctors’ Hospital, 11257 Athens, Greece; sotiris@sgavriil.gr

**Keywords:** metabolic syndrome, hypertension, renal disease, albuminuria, triglycerides, eGFR

## Abstract

*Background*: The influence of metabolic syndrome (MetS) on kidneys is related to many complications. We aimed to assess the association between MetS and chronic renal disease defined by a poor estimated glomerular filtration rate (eGFR) and/or the presence of microalbuminuria/macroalbuminuria. Methods: 149 patients (77 males/72 females) were enrolled in the study. Chronic renal disease was defined according to KDIGO 2012 criteria based on eGFR category and classified albuminuria. MetS was studied as a dichotomous variable (0 to 5 components) including hypertension, waist circumference, low HDL-cholesterol, high triglycerides, and high glucose. *Results*: The association between clustering MetS and both classified eGFR and classified albuminuria (x^2^ = 50.3, *p* = 0.001 and x^2^ = 26.9, *p* = 0.003 respectively) was found to be significant. The MetS presence showed an odds 5.3-fold (1.6–17.8) higher for low eGFR and 3.2-fold (1.2–8.8) higher for albuminuria in combination with the presence of diabetes mellitus, which also increased the risk for albuminuria by 3.5-fold (1.1–11.3). Albuminuria was significantly associated with high triglycerides, hypertension, high glucose (x^2^ = 11.8, *p* = 0.003, x^2^ = 11.4, *p* = 0.003 and x^2^ = 9.1, *p* = 0.01 respectively), and it was mildly associated with a low HDL-C (x^2^ = 5.7, *p* = 0.06). A significant association between classified eGFR and both high triglycerides and hypertension (x^2^ = 9.7, *p* = 0.04 and x^2^ = 16.1, *p* = 0.003 respectively) was found. *Conclusion*: The clustering of MetS was significantly associated with chronic renal disease defined by both classified eGFR and albuminuria. The definition of impaired renal function by classified albuminuria was associated with more MetS components rather than the evaluation of eGFR category. MetS may contribute to the manifestation of albuminuria in patients with diabetes mellitus.

## 1. Introduction

The metabolic syndrome (MetS) is characterized by a combination of metabolic disorders which increase the risk for heart disease, stroke, and all-cause mortality in the general population [1]. These metabolic disorders include central obesity, dyslipidemia (high triglycerides and low HDL-cholesterol), elevated blood pressure and dysregulated glucose homeostasis. Commonly, MetS is defined by the presence of at least three of the above components, but the presence of an increased number of its components confers a much better MetS definition [2,3]. However, some authors have suggested that the relevant risk of cardiovascular disease related to the presence of MetS is no greater than the contribution of the individual components [4]. Previous studies using different populations have reported an elevated age-adjusted prevalence of MetS in relation to increasingly sedentary lifestyle and high rates of obesity [5,6,7].

Chronic kidney disease (CKD) defined by a poor estimated glomerular filtration rate (eGFR) below 60 mL/min/1.73 m^2^ and/or the presence of albuminuria shows also a worldwide increased age-adjusted prevalence rate [8,9]. The influence of MetS on kidneys is related to many complications, but the predominant manifestation is chronic renal failure and the end-stage renal disease as a consequence. Previous observational studies have reported an independent association between MetS and CKD concluding mostly a positive association and less a non-significant correlation [10,11]. It has been supported that patients with MetS have a 1.4, 2.4, or 2.6-fold greater odds of incident CKD (development of an eGFR < 60 mL/min/1.73 m^2^) [12,13,14] than individuals without any MetS components. However, a few studies have been reported to the relationship between MetS and urinary protein excretion [10,15]. On the other hand, individuals with CKD have higher prevalence rates of MetS components than individuals without CKD including traditional and non-traditional predictors, insulin resistance, and elevation of inflammatory markers including cytokines and high-sensitivity C-reactive protein (hsCRP) [3,16].

In this study, we aimed to assess the association between MetS and chronic renal disease defined by an eGFR < 60 mL/min/1.73 m^2^ for a duration of more than 3 months and/or the presence of microalbuminuria/macroalbuminuria in relation to covariates including the age, gender, current smoking, alcohol intake, physical activity, and the presence of diabetes mellitus.

## 2. Materials and Methods

### 2.1. Subjects

This is a single-center observational cross-sectional study which was designed to evaluate the relationship between prevalent components of MetS and chronic renal disease among patients from the Department of Nephrology outpatient clinic of our hospital. This study was conducted in the Private “DOCTORS’ Hospital” and it was approved by the Hospital Institutional Review Board. All study participants provided informed oral consent prior to study enrollment.

One hundred forty nine patients, seventy-seven males, and seventy-two females were enrolled in the study on mean aged 69.4 ± 14.6 years old.

We excluded uncooperative patients and those younger than 18 years old. Subjects with psychiatric symptomatology or established dementia diagnosed by neuropsychologists were also excluded from the study.

The underlying diseases for the follow up in the nephrology outpatient clinic included hypertension (50.3%), type 2 diabetes mellitus (24.2%), chronic glomerulonephritis (5.4%), interstitial nephritis (5.4%), polycystic renal disease (2.7%), and other/unknown (12.1%). Detailed individual medical histories, family histories of kidney diseases, and the current pharmaceutical therapy were obtained from the patients.

Interviews were used to collect demographic data, including age, gender, marital status, education level and lifestyle characteristics regarding with alcohol drinking, active and passive smoking habits and physical activity. Subjects who declared no smoking or alcohol consumption during the past month were considered non-smokers or non-drinkers. Physical activity was measured based on the World Health Organization (WHO) recommendations for healthy adults and physically inactive participants (exercise < 300 min per week) were considered sedentary [17].

The current pharmaceutical treatment mainly included anti-hypertensive medications, such as calcium channel blockers, beta-blockers, inhibitors of angiotensin II AT1 receptors or new central-acting, hypolipidemic and anti-diabetic therapy. The participants consumed a free regular diet using low or regular salt intake.

Anthropometric measurements including height, body weight and waist circumference were recorded. Body mass index (BMI) was calculated and categorized based on the WHO classification into underweight (<18.5 kg/m^2^), normal weight (18.5–24.9 kg/m^2^), overweight (25–29.9 kg/m^2^) and obese (≥30 kg/m^2^) [18]. Waist circumference measurements were made approximately at the midpoint between the lower margin of the last palpable rib and the top of iliac crest at the end of a normal expiration according to the WHO guidelines [19].

### 2.2. Biochemical Measurements

Fasting plasma glucose, creatinine, triglycerides, high-density lipoprotein-cholesterol (HDL-C) were recorded from the patient files using the latest results. These biochemical markers were measured using a spectrophotometric technique by Chemistry Analyzer (MINDRAY BS-200, Diamond Diagnostics, Massachusetts, MA, USA) and were represented as mg/dL.

Spot urine samples from the first micturition after rising were obtained for the measurement of albumin and creatinine concentrations by the Chemistry Analyzer. The urinary albumin analysis by a spot urine sample is more accurate than dipsticks, but this still generates a concentration than a quantity.

### 2.3. Blood Pressure Measurements—Definitions

Blood pressure data were considered over a month, as the exposure period.

We recommended the home blood pressure record as a peripheral systolic and diastolic blood pressure (SBP and DBP) measurement using an automatic sphygmomanometer OMRON M4-I (Co Ltd., Kyoto, Japan). The blood pressure was doubly measured for two times per day, in the morning after rising and in the evening in a fasting, calming and resting state and two means were recorded per day. Their average was used for statistical analysis. Mean peripheral blood pressure (MBP) was calculated as: MBP = DBP + 1/3(SBP − DBP).

We also used a 24-h ambulatory blood pressure monitor with the Mobil-O-Graph device for verification of measurements and whether the means blood pressure values significantly differed from home recorded values, the means by 24-h monitoring were used for statistical analysis rather than the means by home measurements.

Chronic renal disease was defined according to the Kidney Disease Improving Global Outcomes (KDIGO) 2012 criteria as eGFR < 60 mL/min/1.73 m^2^ for time duration more than 3 months and the participants were classified in five stages according to eGFR category [20]. The eGFR was calculated using the Chronic Kidney Disease Epidemiology Collaboration (CKD-EPI) equation and the Modification of Diet in Renal Disease (MDRD) equation. The classification of our subjects based on eGFR category included: G1 eGFR ≥ 90 mL/min/1.73 m^2^ (*n* = 8, 5.4%); G2 eGFR = 60–90 mL/min/1.73 m^2^ (*n* = 34, 22.8%); G3 eGFR = 30–60 mL/min/1.73 m^2^ (*n* = 76, 51%); G4 eGFR = 15–30 mL/min/1.73 m^2^ (*n* = 29, 19.5%); G5 eGFR < 15 mL/min/1.73 m^2^ (*n* = 2, 1.3%).

The enrolled subjects were also classified based on albuminuria, which was defined as urinary albumin-to-creatinine ratio (ACR) ≥ 30 mg/gr according to KDIGO 2012 [20]. ACR calculation by using a spot urine sample, such as in this study, is considered an acceptable calculation, as ACR is correlated well with 24-h urinary albumin excretion. The distribution of albuminuria in our data included the three stages: A1 ACR < 30 mg/gr (normal, *n* = 51, 34.2%); A2 ACR 30–300 mg/gr (microalbuminuria, *n* = 69, 46.3%) and A3 ACR > 300 mg/gr (macroalbuminuria/proteinuria, *n* = 29, 19.5%).

We used the International Diabetes Federation (IDF) criteria for MetS diagnosis [21]. Three or more of the following criteria were used for the diagnosis of MetS: systolic blood pressure (SBP) ≥ 130 mmHg and/or diastolic blood pressure (DBP) ≥ 85 mmHg and/or with pre-existed individual history of hypertension and/or with taking antihypertensive therapy, central obesity defined by a waist circumference ≥ 94 cm in males and ≥80 cm in females, triglycerides ≥ 150 mg/dL or specific treatment for this lipid abnormality, HDL-C < 40 mg/dL in males and <50 mg/dL in females and fasting plasma glucose ≥ 100 mg/dL or previously diagnosed type 2 diabetes mellitus. The prevalence of MetS in our data defined by more than three characteristics was equal to 80.5% (*n* = 120). The distribution of MetS components included: without any MetS component *n* = 2, with one MetS component *n* = 4, with two MetS components *n* = 23, with three MetS components *n* = 36, with four MetS components *n* = 44 and with five MetS components *n* = 40.

## 3. Data Analysis

Data were analyzed using SPSS 15.0 statistical package for Windows (SPSS Inc., Chicago, IL, USA) and expressed as mean ± standard deviation or as median value ± inter-quartile range for data that showed skewed distribution. Differences between mean values were assessed by using an unpaired *t*-test for two groups and data that showed skewed distributions were compared with Mann-Whitney *U*-test.

Correlations between categorical variables were defined by chi-square tests. *p* values less than 0.05 were considered significant. We built models using logistic regression analysis showing the risk for the development of low eGFR and albuminuria by the presence of MetS components adjusting to the age, gender, current smoking, alcohol taking, physical activity and the presence of diabetes mellitus.

## 4. Results

The Socio-demographical and biochemical characteristics of our data are shown in Table 1 and Table 2.

In Table 3 the differences between the subjects with and without MetS are shown. We observed that the patients with MetS were older and they had significantly higher BMI, ACR, triglycerides, glucose, waist circumference, but significantly lower HDL-C and eGFR than the patients without MetS.

**Figure 1:**
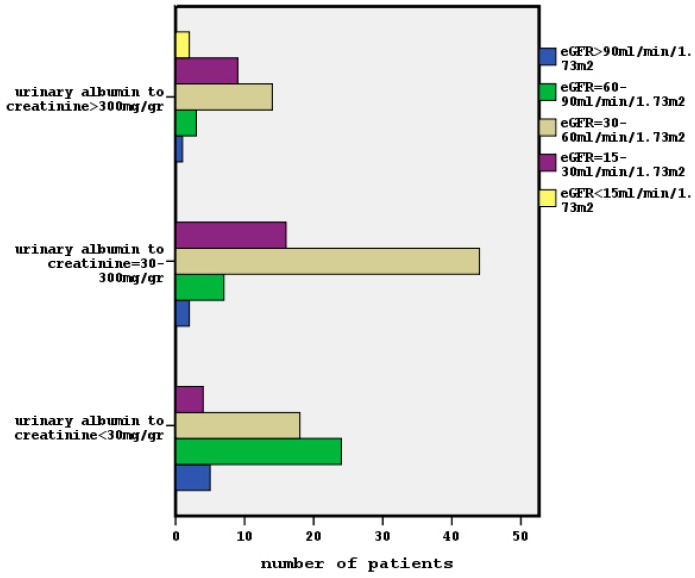
The association between classified eGFR and classified albuminuria in our data (x^2^ = 41.8, *p* = 0.001).

The relationship between classified eGFR and albuminuria is shown in Figure 1 (x^2^ = 41.8, *p* = 0.001). Five of our participants had an eGFR > 90 mL/min/1.73 m^2^ and an ACR < 30 mg/gr (G1 and A1 stages) meaning a normal kidney function.

**Figure 2:**
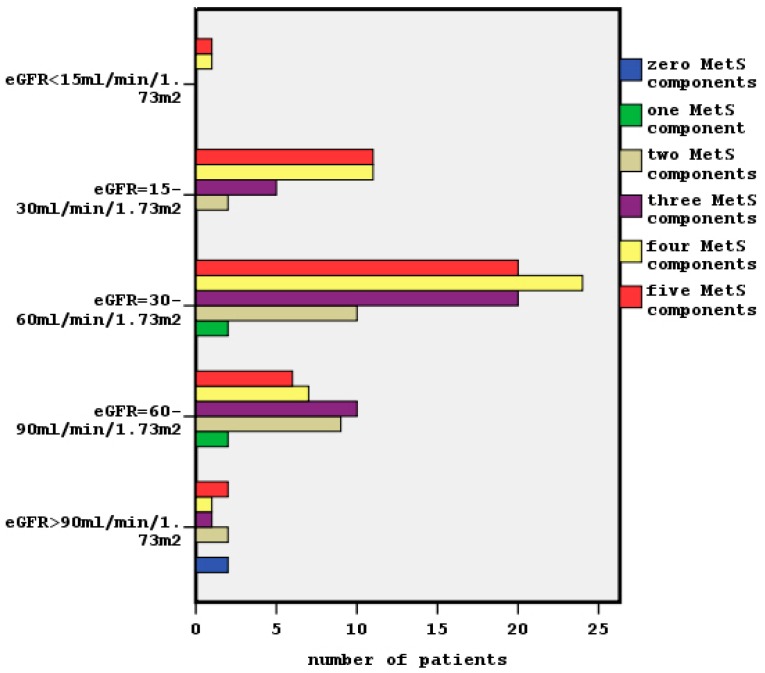
The relationship between clustering of MetS and classified eGFR (x^2^ = 50.3, *p* = 0.001).

Chi-square tests showed a significant association between clustering MetS components and both classified eGFR and classified albuminuria (x^2^ = 50.3, *p* = 0.001, Figure 2 and x^2^ = 26.9, *p* = 0.003 respectively).We observed that the most of patients in G3 and G4 stages had four and five MetS components, although in G2 stage the presence of two and three MetS components was predominant. Similarly, the distribution of increased MetS components was shown in A2 and A3 stages of albuminuria. The two patients without any MetS characteristic were in stages G1 and A1, so they had normal kidney function.

**Figure 3:**
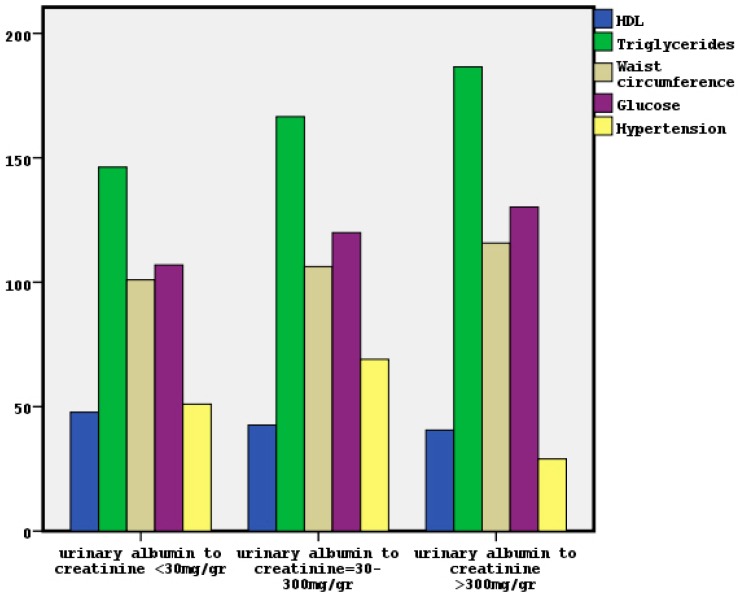
The distribution of MetS components in classified albuminuria.

In Figure 3 the nomination of MetS characteristics in classified albuminuria is depicted. We noted significant association between classified albuminuria and high triglycerides, hypertension and high glucose by x^2^ tests (x^2^ = 11.8, *p* = 0.003, x^2^ = 11.4, *p* = 0.003 and x^2^ = 9.1, *p* = 0.01 respectively). The relationship between classified albuminuria and low HDL-C trended to be significant (x^2^ = 5.7, *p* = 0.06), although the association with waist circumference was found non-significant. On the other hand, we observed a significant association between classified eGFR and both high triglycerides and hypertension (x^2^ = 9.7, *p* = 0.04 and x^2^ = 16.1, *p* = 0.003 respectively). The association between classified eGFR and low HDL-C, high waist circumference or high glucose was found non-significant.

Moreover, x^2^ tests showed a significant association between the presence of MetS and the cause of renal disease, female gender and positive family history for cardiovascular events (x^2^ = 17.8, *p* = 0.003, x^2^ = 8.4, *p* = 0.003 and x^2^ = 7.3, *p* = 0.003 respectively). We could observe that everybody of diabetic patients had manifested MetS, although a few hypertensive subjects (*n* = 15) had not MetS. The association between MetS and smoking, alcohol taking and physical activity was found non-significant.

Table 4 and Table 5 show the risk factors for low eGFR and manifestation of albuminuria. The presence of MetS showed an odds 5.3-fold (1.6–17.8) higher for a low eGFR and 3.2-fold (1.2–8.8) higher for albuminuria adjusting to the age, gender, smoking, alcohol intake, physical activity and the presence of diabetes mellitus. The old age and female gender were also found to be significant risk factors for the low eGFR, although the presence of diabetes mellitus showed a risk 3.5-fold (1.1–11.3) higher for manifested albuminuria in combination with clustering of MetS.

## 5. Discussion

In this cross-sectional study, we aimed to examine the associations between metabolic components and (i) classified chronic renal disease based on eGFR category and (ii) classified albuminuria using the MetS as a dichotomous variable (0 to 5 components) in a population of patients who were followed in the outpatient clinic of a Department of Nephrology in Greece. We also evaluated the role of underlying disease including diabetes mellitus and socio-demographic characteristics.

In our data, the prevalence of a low eGFR (<60 mL/min/1.73 m^2^) was found in 71.8% of patients and the presence of microalbuminuria/macroalbuminuria in 65.8%. A few of our subjects had microalbuminuria/macroalbuminuria (*n* = 13) despite having an eGFR > 60 mL/min/1.73 m^2^ (G1 and G2 stages), as depicted in Figure 1. Conversely, some of our participants with a poor eGFR (*n* = 22) did not have albuminuria. Indeed, the kidney damage defined by the presence of albuminuria (ACR > 30 mg/gr) can be combined by a normal or raised GFR, in agreement with previous reports [9]. Only five of our enclosed subjects had a normal renal function determined by an ACR < 30 mg/gr in conjunction with an eGFR > 90 mL/min/1.73 m^2^.

The adjusted association between MetS and both low eGFR and the presence of albuminuria was significant in our data using, as covariates, the age, gender, current smoking, alcohol intake, physical activity, and the presence of diabetes mellitus. The presence of MetS showed an odds 5.3-fold (1.6–17.8) higher for the low eGFR in combination with the old age and female gender and a risk 3.2-fold (1.2–8.8) higher for albuminuria in combination with the presence of diabetes mellitus. In agreement, previous studies reported an elevated risk for CKD due to the presence of MetS [14,15].

The presence of diabetes mellitus showed an odds 3.5-fold (1.1–11.3) higher in our adjusted model for the prediction of albuminuria in conjunction with MetS, but not for a low eGFR prediction. MetS may contribute to the manifestation of albuminuria in patients with diabetes mellitus, but it does not contribute to an impaired glomerular filtration rate in diabetic patients. However, there is a debate about the role of MetS on the overall risk of patients with diabetes mellitus. Previously, it has been reported that inflammatory markers related to cardiovascular disease did not significantly differ in subjects with and without MetS [22]. On the other hand, albuminuria in diabetics may be associated with comorbidities due to diabetes mellitus [23,24].

Moreover, according to our findings, the presence of MetS in the elderly population may be associated with an impaired eGFR in these patients (mainly female) rather than with manifested albuminuria. Smoking and alcohol were not found to be significant risk factors for a poor eGFR or albuminuria in our data, despite their known association with CKD [3].

In the meantime, in this study, we observed that the patients who had advanced chronic renal disease (G3, G4 and G5 stages) presented with more MetS components (mainly 4 and 5 components) than the patients in the earlier chronic renal disease stages (whom had 2 or 3 MetS components) as it is shown in Figure 2. The findings for the prevalence of dichotomous MetS on microalbuminuria/macroalbuminuria were similar.

In agreement, it has previously been supported that MetS, when studied by number of components rather than by a single component, was a significant independent risk factor for CKD defined by eGFR < 60 mL/min/1.73 m^2^ [3]. Individuals with up to five components present had increased odds of developing CKD, as opposed to those without any components present, due perhaps to an additive detrimental effect by the cluster of MetS components rather than by single components [10,13].

Furthermore, the unadjusted association between classified albuminuria and separated MetS components showed that high triglycerides, high glucose, manifested hypertension and low HDL were significant factors, although only the high triglycerides and hypertension were found to be important factors for classified eGFR. As albuminuria determines the presence of underlying kidney damage, even with normal or high eGFR, the findings related to the CKD classification based on albuminuria may be more valid to define the role of metabolic abnormalities on kidney function, in comparison to the findings regarding classified eGFR.

In accordance, previously it has been reported that hypertriglyceridemia was associated with an increased risk of CKD development, and that high triglycerides were the main risk factor for proteinuria [25,26].

The relationship between MetS and CKD should be biologically plausible, but the pathophysiological mechanisms of MetS-induced kidney disease are not fully understood. Visceral obesity and adipose tissue expansion, which accompany the MetS, are highly correlated with insulin resistance [27]. Adipose tissue promotes chronic inflammation and oxidative stress that exacerbate insulin resistance. Insulin resistance is increasingly becoming recognized as a non-traditional risk factor for CKD because it is linked with the causal pathway to CKD rather than inflammation [3,28]. Insulin resistance and inflammation are associated with endothelial dysfunction, reduced synthase of endothelial nitric oxide, and worsening of renal hemodynamic function in conjunction with injury of podocytes resulting in hypertension and albuminuria [29,30]. Moreover, it has been reported that insulin resistance is associated with sodium retention, overproduction of low-density lipoprotein cholesterol, and hypertriglyceridemia, which may impair mitochondrial function and promote kidney cell damage [31].

Indeed, despite not evaluating insulin resistance in the present study, we noted that the subjects with MetS had significantly higher BMI and waist circumference, perhaps implicating a higher insulin resistance, in combination with significantly higher triglycerides, glucose, and ACR. However, they were found to have significantly lower HDL and eGFR than the patients without MetS.

Furthermore, the role of insulin resistance linked with the MetS establishment may explain the observed significant association between the MetS presence and the cause of renal disease. We noted that some patients with hypertensive nephrosclerosis had not manifested MetS, although the total of diabetic participants had MetS, due perhaps to increasingly insulin resistance in diabetics rather than in hypertensive patients.

Hypertension and diabetes mellitus and their combination may be associated with an increased prevalence of CKD [32,33]. Indeed, in our data, hypertension was recorded as the main cause of chronic renal disease. Type 2 diabetes mellitus was recorded as the secondary cause in agreement with recent previous study [14].

In this study, we also observed that the definition of MetS was significantly associated with female gender, in agreement with previous studies [14,34]. The reasons for this finding may include a complex of hormonal and socio-cultural interactions that predispose females to demonstrate more MetS components than males.

The relationship between MetS and positive family history for cardiovascular events was found to be significant, although the associations with smoking habits, alcohol intake and physical inactivity were found to be non-significant in this data. The association between alcohol consumption and MetS is controversial. Studies have shown a positive association and others have observed a negative association or no relation at all, as is the case in our study [35,36]. The controversy is attributed to a complicated relationship between alcohol consumption and each component of MetS.

Mild to moderate alcohol consumption may have a favorable influence on lipid metabolism, central obesity and glucose regulation [37], but, on the other hand, alcohol causes hypertension [38] and hypertriglyceridemia [39], resulting in alcohol-related MetS.

As the clustering of MetS was significantly associated with both classified eGFR and classified albuminuria in this study, bigger studies need to clarify the role of mild alcohol consumption on MetS outcomes in patients with impaired renal function. However, the change of lifestyle including physical activity, better nutrition and the avoidance of obesity should be very beneficial to adverse the intensive MetS and prevent the progression of cardiovascular and renal disease.

## 6. Limitations

The main limitation of this study is the cross-sectional nature of the study performed in a single-center: the Department of Nephrology. Also, the measurement of albumin using spot urine confers a concentration rather than a quantitative measurement.

## 7. Conclusions

The clustering of MetS was significantly associated with chronic renal disease defined by both classified eGFR and albuminuria. The definition of impaired renal function by the classification based on albuminuria was associated with more MetS components rather than the evaluation of eGFR category. MetS may contribute to the manifestation of albuminuria in patients with diabetes mellitus and to a poor eGFR in the elderly female population.

## Figures and Tables

**Table 1:** Socio-demographical characteristics of studied patients (*n* = 149).

Characteristic	Frequency in Number of Patients
Gender (males/females)	77/72
BMI (<25/>25)	12/137
Waist circumference (<80 or 94/>80 or 94)	11/138
Hypertension (yes/no)	132/17
Smoking (yes/no)	29/120
Alcohol intake (yes/no)	42/107
Physical activity (yes/no)	49/100
Family history of cardiovascular events (yes/no)	116/33
Prevalence of MetS (yes/no)	120/29
Clustering of MetS (0 to 5 components)	2/4/23/36/44/40
Underlying disease:	
diabetes mellitus	*n* = 36
Hypertension	*n* = 75
Chronic glomerulonephritis	*n* = 8
Interstitial nephritis	*n* = 8
Polycystic disease	*n* = 4
Other/unknown	*n* = 18

Smoking (yes): smoking habits in the past month; Alcohol intake (yes): alcohol consumption in the past month; Physical activity (yes): exercise > 300 min/week; BMI: Body mass index.

**Table 2:** Biochemical characteristics of studied patients (*n* = 149).

Characteristic	Frequency in Number of Patients
HDL-C (>40 or 50/<40 or 50)	72/77
Triglycerides (<150/>150)	60/89
Glucose (<100/>100)	50/99
ACR (<30/>30 mg/gr)	51/98
Classified albuminuria:	
A1: ACR < 30 mg/gr	*n* = 51
A2: ACR = 30–300 mg/gr	*n* = 69
A3: ACR > 300 mg/gr	*n* = 29
eGFR < 60 mL/min/1.73 m^2^ (yes/no)	107/42
Classified eGFR:	
G1: eGFR > 90 mL/min/1.73 m^2^	*n* = 8
G2: eGFR = 60–90 mL/min/1.73 m^2^	*n* = 34
G3: eGFR = 30–60 mL/min/1.73 m^2^	*n* = 76
G4: eGFR = 15–30 mL/min/1.73 m^2^	*n* = 29
G5: eGFR <15 mL/min/1.73 m^2^	*n* = 2

HDL-C: high density lipoprotein-cholesterol; ACR: albumin-to-creatinine ratio in urine sample; eGFR: estimated glomerular filtration rate.

**Table 3:** Differences between groups of patients with (*n* = 120) and without (*n* = 29) metabolic syndrome (MetS) defined by more than three components.

Characteristic	Patients with MetS (n = 120) Mean ± SD/Mean Rank	Patients without MetS (n = 29) Mean ± SD/Mean Rank	*p* Value
Sex (%males/%females)	55 (45.8%)–65 (54.2%) *	22(75.9%)–7 (24.1%)	0.003
Age (years)	70.8 ± 13.5	64.0 ± 18.05	0.06
BMI (Kg/m^2^)	/81.1 *	/49.5	0.001
eGFR (mL/min/1.73 m^2^)	45.1 ± 19.2 *	58.4 ± 23.7	0.002
Classified eGFR: *			
G1: eGFR > 90 mL/min/1.73 m^2^	4 (50%)	4 (50%)	
G2: eGFR = 60–90 mL/min/1.73 m^2^	23 (67.6%)	11 (32.4%)	
G3: eGFR = 30–60 mL/min/1.73 m^2^	64 (84.2%)	12 (15.8%)	0.01
G4 :eGFR = 15–30 mL/min/1.73 m^2^	27 (93.1%)	2 (6.9%)	
G5 :eGFR < 15 mL/min/1.73 m^2^	2 (100%)	0 (0%)	
ACR (mg/gr)	/79.6 *	/55.8	0.008
*Classified albuminuria:* *			
A1: ACR < 30 mg/gr	34 (66.7%)	17 (33.3%)	
A2: ACR = 30–300 mg/gr	59 (85.5%)	10 (14.5%)	0.006
A3: ACR > 300 mg/gr	27 (93.1%)	2 (6.9%)	
Underlying disease: *			
- diabetes mellitus	36 (100%)	0 (0%)	
- Hypertension	60 (80%)	15 (20%)	
- Chronic glomerulonephritis	6 (75%)	2 (25%)	
- Interstitial nephritis	5 (62.5%)	3 (37.5%)	0.003
- Polycystic disease	3 (75%)	1 (25%)	
- Other/unknown	10 (55.6%)	8 (44.4%)	
Family cardiovascular history (yes/no)	88 (73.3%)/32 (26.7%) *	28 (96.6%)/1 (3.4%)	0.003

BMI: Body mass index; HDL-C: high density lipoprotein-cholesterol; eGFR: estimated glomerular filtration rate; ACR: albumin-to-creatinine ratio in urine sample; *: *p* < 0.05.

**Table 4:** Logistic regression analysis showing the predictors for the manifestation of a poor estimated glomerular filtration rate, eGFR < 60 mL/min/1.73 m^2^ (*n* = 149).

Characteristic	*p*-Value	Odds Ratio	Confidence Interval
Age *	0.001	1.07	1.04–1.11
Gender *	0.02	0.3	0.1–0.9
smoking	0.2	0.4	0.1–1.3
Alcohol intake	0.9	0.9	0.3–2.7
Physical activity	0.5	0.7	0.3–1.9
presence of MetS *	0.006	5.3	1.6–17.8
Diabetes mellitus	0.9	0.93	0.3–2.8

Smoking (yes): smoking habits in the past month; Alcohol intake (yes): alcohol consumption in the past month; Physical activity (yes): exercise > 300 min/week; *: *p* < 0.05.

**Table 5:** Logistic regression analysis showing the predictors for the manifestation of albuminuria (albumin-to-creatinine ratio in urine sample, ACR > 30 mg/gr) (*n* = 149).

Characteristic	*p*-Value	Odds Ratio	Confidence Interval
age	0.07	1.02	0.9–1.05
gender	0.1	0.5	0.2–1.1
smoking	0.9	0.95	0.3–2.6
Alcohol intake	0.4	1.5	0.6–3.9
Physical activity	0.3	0.6	0.2–1.5
presence of MetS *	0.02	3.2	1.2–8.8
Diabetes mellitus *	0.03	3.5	1.1–11.3

Smoking (yes): smoking habits in the past month; Alcohol intake (yes): alcohol consumption in the past month; Physical activity (yes): exercise > 300 min/week. *: *p* < 0.05.
